# Root-Associated Mycobiomes of Common Temperate Plants (*Calluna vulgaris* and *Holcus lanatus*) Are Strongly Affected by Winter Climate Conditions

**DOI:** 10.1007/s00248-020-01667-7

**Published:** 2021-01-16

**Authors:** Mathilde Borg Dahl, Derek Peršoh, Anke Jentsch, Jürgen Kreyling

**Affiliations:** 1 Institute of Microbiology, Center for Functional Genomics of Microbes, University of Greifswald, Felix-Hausdorff-Str. 8, 17487 Greifswald, Mecklenburg-Vorpommern Germany; 2 Geobotanik, Ruhr-Universität Bochum, Bochum, Germany; 3 Disturbance Ecology, Bayreuth Center of Ecology and Environmental Research BayCEER, University of Bayreuth, Bayreuth, Germany; 4 Institute of Botany and Landscape ecology, University of Greifswald, Greifswald, Germany

**Keywords:** Climate change, *Calluna vulgaris*, DNA barcoding, EVENT experiments, *Holcus lanatus*, Plant-fungi associations, Root associated mycobiome

## Abstract

Winter temperatures are projected to increase in Central Europe. Subsequently, snow cover will decrease, leading to increased soil temperature variability, with potentially different consequences for soil frost depending on e.g. altitude. Here, we experimentally evaluated the effects of increased winter soil temperature variability on the root associated mycobiome of two plant species (*Calluna vulgaris* and *Holcus lanatus*) at two sites in Germany; a colder and wetter upland site with high snow accumulation and a warmer and drier lowland site, with low snow accumulation. Mesocosm monocultures were set-up in spring 2010 at both sites (with soil and plants originating from the lowland site). In the following winter, an experimental warming pulse treatment was initiated by overhead infrared heaters and warming wires at the soil surface for half of the mesocosms at both sites. At the lowland site, the warming treatment resulted in a reduced number of days with soil frost as well as increased the average daily temperature amplitude. Contrary, the treatment caused no changes in these parameters at the upland site, which was in general a much more frost affected site. Soil and plant roots were sampled before and after the following growing season (spring and autumn 2011). High-throughput sequencing was used for profiling of the root-associated fungal (ITS marker) community (mycobiome). Site was found to have a profound effect on the composition of the mycobiome, which at the upland site was dominated by fast growing saprotrophs (Mortierellomycota), and at the lowland site by plant species-specific symbionts (e.g. *Rhizoscyphus ericae* and *Microdochium bolleyi* for *C. vulgaris* and *H. lanatus* respectively). The transplantation to the colder upland site and the temperature treatment at the warmer lowland site had comparable consequences for the mycobiome, implying that winter climate change resulting in higher temperature variability has large consequences for mycobiome structures regardless of absolute temperature of a given site.

**Supplementary Information:**

The online version contains supplementary material available at 10.1007/s00248-020-01667-7.

## Introduction

Winter soil temperature is an important driver for many ecological and biogeochemical processes [1] and was reported as a predictor for plant richness and phenology in cold temperate and boreal terrestrial ecosystems [2, 3]. Climate change is increasing the average winter air temperatures in many regions of the temperate zone, with consequences for stability and length of snow cover and subsequent consequences for soil insulation [4–6]. However, frost events are predicted to occur with unchanged magnitude and duration as nowadays in many temperate regions [7]; therefore, winter air and soil temperatures are expected to become more variable [8, 9]. The increased variance in soil temperature will likely increase the frequency of soil frost and freeze–thaw cycles, which can physically damage plant roots [10], break up soil aggregates [11] and lyse microbial cells through physical and osmotic stress [12, 13]. For warmer lowland temperate regions, however, although soil temperature variability might increase, an increase in winter temperatures could generally lead to fewer frost events (e.g. lowland Germany [2, 5]). Contrasting effects of winter climate change can therefore be expected for colder versus warmer temperate regions [14]. For these reasons, a temperature manipulation experiment was set up under two conditions (at a relatively warm lowland site and a colder upland site, see the ‘Methods’ section). Two common temperate plant species, *Calluna vulgaris* and *Holcus lanatus*, were planted in monoculture mesocosms and winter soil temperature was experimentally manipulated (induced by short winter warming pulses, for details, see the “Methods” section and Schuerings et al. (2014b) [15]).

Both plant species are known to form mycorrhizal associations (ericoid and arbuscular respectively for *C. vulgaris* and *H. lanatus*). Such symbioses and other plant properties e.g. exudates and root morphology [16] are known to shape rhizobiome microbial assembly [17]. Therefore, it is essential to also consider the response of the associated microorganisms when studying plant response to climate change. In this article, we report the results from an investigation of the soil fungal community (mycobiome from hereon) associated with the plant roots in the above described experiment.

It was previously reported that the warming pulses significantly reduced the snow cover at both experimental sites (lowland and upland) and increased variability of soil temperatures, but not significantly affecting the number of freeze-thaw cycles (8/7 and 5/6 cycles across winter 2010/2011 for ambient/warming treatment at the lowland and upland site respectively) [18]. Likewise, a number of plant traits were previously reported from the experiment [15, 18]; the main trends from these studies are summarized in Table 1. In general, both plant species (which were pre-grown under the same greenhouse conditions prior to the experiment) had a larger above-ground biomass (end of growing season) at the upland site, and an increased nitrogen (N) availability and enzymatic activity was measured in the soil throughout the winter at this site (Table 1). The winter warming pulse treatment consistently increased the microbial activity (measured by bait-lamina sticks) across sites, but had inconsistent effects on the plant responses at both sites.

**Table 1:**
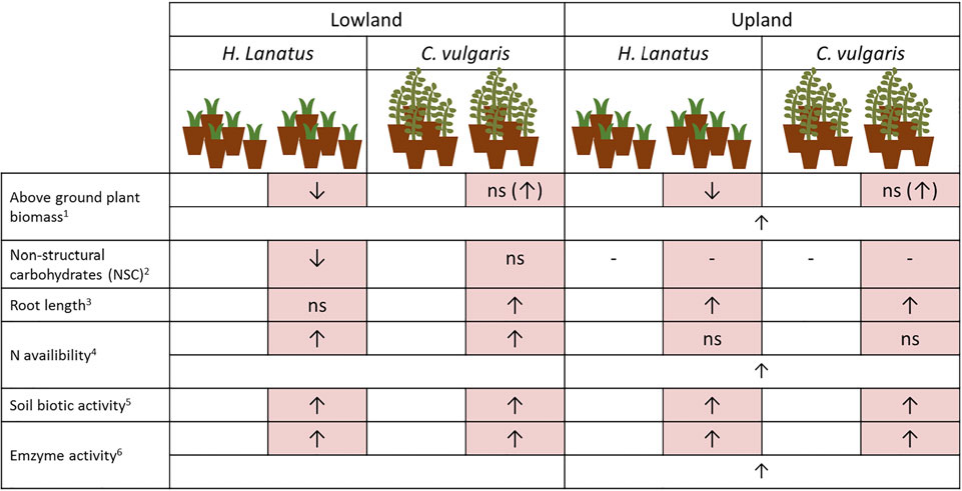
Overview of the main trends from previously published results of the experiment. Red columns indicate warming treatments. Arrows indicate the directional change relative to ambient plots. Cross-treatment bars indicate site-specific effects. ns, non-significant. For further details, see Schuerings et al. (2014a), 2014b [15, 18]. All measurements were taken at multiple time points in the winter 2010/2011, except biomass which was measured at the end of the growing season 2011 and 2012. ^1)^For *H. lanatus* harvested completely twice a year for *C. vulgaris*, biomass was estimated based on biometric measurements and calibrated against harvested individuals. ^2)^Measured from leaf material and only measured at the lowland site. NSC is cryoprotective structures (lowering freezing point of the plant tissue). ^3)^Root length was acquired via minirhizotron technique in a clear plastic tube monitoring root development. ^4)^Plant-available N measured with resin stick method install in each mesocosm during the winter. ^5)^Measured with bait-lamina sticks containing 16 baits which were inserted vertically in the top soil layer of every mesocosm prior to the warming pulses treatment. ^6)^Potential extracellular enzymatic activity measured from soil extractions with assays for beta-glucosidase, cellobiohydrolase, acid phosphatase, xylosidase (the latter two showed only site-specific differences)

Many microbial organisms are sensitive to frost-stress [13, 19] with documented consequences for microbial associated processes, such as nitrogen mineralisation [20, 21]. Fungi are in general hypothesised to be more frost sensitive than other soil microbes, due to their filamentous growth which may be more susceptible to frost damage than e.g. single-celled organisms [13, 22]. Damage to mycorrhizal fungi may have a particularly strong impact on plant communities and their productivity. Klironomos et al. (2001) [23] found that freezing treatment reduced the per cent colonization of five tested arbuscular mycorrhizal (AM) fungi on five common northern temperate plant species, but also that species-specific responses existed which could be linked to life strategy and morphology of the fungal species. Contrary, Lekberg et al. (2008) [24] found no indications of local adaptation among globally distributed AM fungi in their response to freezing. Finally, examples of microbes mitigating frost-stress in plants also exists [25] and the concept is applied in agricultural production where plants are inoculated with specific microbes with the ability to influence plants’ biochemistry [17, 25–29].

Based on these previous findings (Table 1), we expect the mycobiome community composition to have shifted in response to the warming treatment which has generally led to an increased below ground activity (enzyme- and biotic activity, N availability and root growth). Changes in the mycobiome are likely driver of the increased N availability and we expect both saprotrophic and mycorrhizal fungi to show altered abundances patterns under ambient and warming conditions.

## Methods

### Experimental Design and Sampling

The experimental design was previously described in Schuerings et al. (2014b) [15]. In brief, plant seedlings of the grass *Holcus lanatus* and 2-year-old plants of the shrub *Calluna vulgaris* (juvenile plants randomly picked from the same cohort, both pre-grown under controlled greenhouse condition at the lowland site) were planted in May 2010 in monoculture mesocosms (barrels; 50 cm diameter and 80 cm depth) which were then set up at two sites; the lowland site located in the Ecological-Botanical Garden of the University of Bayreuth, Germany (49° 55′ 36.32″ N, 11° 34′ 57.28″ E, 358 m a.s.l.) and the upland site located at the Waldstein mountain in the Fichtelgebirge, Germany (50° 8′ 35.81″ N, 11° 51′ 50.92″ E, 781 m a.s.l.). The soil substrate was a natural soil consisting of homogenized loamy sand (77% sand, 16% silt, 7% clay) from a sand quarry nearby the lowland site where both plant species naturally occur, with a pH = 7 and a total carbon content of 2.4%. The barrels were attached with outlet hoses at the bottom of each mesocosm, so that the mesocosms functioned as zero tension lysimeters. In the following winter, warming pulses were administrated between December 2010 and February 2011. Warming pulses were started when weather forecast predicted air frost (at both sites) for at least 48 h in which the warming pulses took place. Each factorial combination (species by warming treatment by site) was replicated five times. Soil (− 2 cm depth) and air temperature (+ 5 cm height; one per treatment and experimental site) were measured hourly by thermistores (B57863-S302-F40, EPCOS) connected to a datalogger (dl2, Delta). The upland site was generally colder, experiencing lower annual air temperatures and higher annual precipitation (Schuerings et al. 2014b [15] and suppl. S1).

**Fig. 1:**
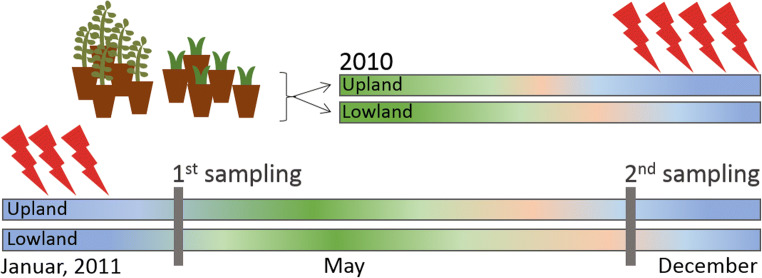
Schematic overview of experimental timeline and sampling. Ten replicates of *Calluna vulgaris* and *Holcus lanatus* were planted and distributed to two sites (five replicates per plant per site) in May 2010; warming pulses (indicated with red arrows) were administrated through-out the winter 2010/2011 and root samples collected on March 14 and December 1 2011 (before and after the 2011 growing season)

The experimental design and sampling is summarized in Fig. 1.

For this study, samples were collected on March 14, 2011, and December 1, 2011 (during plant dormancy before and after the growing season following the warming pulses treatment). From each mesocosm, five soil cores (2 cm diameter) were collected and fine roots were taken from the cores by hand and frozen at − 80 °C.

### DNA Extraction and Library Preparation

A bead mixture (0.03 g of Ø 0.1–0.25 mm, 0.06 g of Ø 0.25–0.5 mm and 5–6 glass beads Ø 1.25–1.55 mm) was added to approximately 1 cm of fine roots for homogenisation in a FastPrep Instrument (MP Biomedicals) at 6 m s^−1^ for 60 s. DNA was extracted using the Charge Switch gDNA Plant Kit (Invitrogen) for details see Kreyling et al. (2012) [30].

An ITS amplicon library was constructed using the fungal specific primers ITS1F [31] and ITS4 [32] as detailed by Peršoh et al. (2018) [33]. Briefly, the 80 samples were multiplexed in two consecutive PCRs (adding unique barcodes per sample), equimolary pooled and purified. Paired-end sequencing (2 × 250 bp; Kit v3 Chemistry) of the amplicon was conducted by the sequencing service of the Faculty of Biology at LMU Munich, using the Illumina MiSeq platform (Illumina Inc.). Raw sequencing files are available from NCBI (BioProject: PRJNA678839).

### Bioinformatic Processing

Using the QIIME pipeline [34] sequence reads were assigned to samples according to the indices and barcodes. Quality filtering was applied using the same command. In agreement with earlier studies, only the forward orientated reads, representing the ITS1 rRNA gene region, were further processed [33, 35, 36]. ITS1 reads of samples with identical barcodes were trimmed at the 5′-end to the final 11 bp of the SSU rRNA gene region and at the 3′-end to a length of 172 bp using the FastX toolkit (www.hannonlab.cshl.edu/fastx_toolkit/). The trimmed ITS1 reads, with retained quality scores, were subjected to CD-HIT-OTU [37] for clustering [38] (www.weizhongli-lab.org/cd-hit-otu/). An OTU (operational taxonomic units) table, which lists the number of reads in each cluster for each sample, was generated by applying a 97% similarity threshold. OTUs were assigned to taxa using QIIME and the UNITE database v7 [39] as reference (The OTU table is available from suppl. S2). The phyla ‘Zygomycota’ was renamed to ‘Mortierellomycota’ for the final publication, following recent nomenclature [40]. Both DNA read-based (individual) and sample-based OTU accumulation curves were constructed following [41] to insure sampling saturation was reached (suppl. S3). Prior to analysis, samples were rarified to 30,000 reads per sample (five samples had less reads but showed saturated OTU accumulation curves, these samples were kept without rarefication). The results of this study were checked on nonrarefied raw data and found to be qualitatively the same without rarefaction (data not shown). The online application FUNGuild [42] was used for functional classification of the fungal community, distinguishing between plant pathogens, mycorrhizal fungi (arbuscular, ecto- and ericoid mycorrhizal) and saprotrophic fungi; the latter was further separated into three categories: (i) purely saprotrophic, (ii) potential symbiotroph (endophytes) and (iii) potential wood degraders.

### Data Analysis and Statistics

Basic statistical analyses were performed in R v. 3.3.3 [43] using mainly the packages ‘vegan’ [44] and ‘iNEXT’ [45]. Analysis scripts are available from supplementary S4. All averages are reported with ± standard deviation, statistical significance differences between means were tested using Kruskal-Wallis test as a consequence of unequal sampling sizes between categories.

For the 59 recovered fungal communities, distance-based redundancy analysis (dbRDA) was performed using Bray-Curtis (BC) dissimilarity matrix of the Hellinger-transformed fungal community with 1000 permutations. Experimental parameters were as follows: site (upland and lowland site), sampling time (spring, fall), plant species (*C. vulgaris, H. lanatus*) and treatment (ambient, warming). PERMANOVA (permutational multivariate analysis of variance [46]) analysis was carried out to calculate interaction effects between the experimental parameters by applying the function ‘adonis2’ from the R package ‘vegan’. Prior to analysis, the dispersion (variance) between groups was tested with the function ‘betadisper’ (R package ‘vegan’) and was found non-significant for all parameters. A heatmap was made of the ten most abundant OTUs, with presence in minimum half of the samples from a given site. Data were log transformed before *z* scores were calculated for improved graphic presentation. OTUs with similar distribution patterns across samples were identified by hierarchical clustering constrained by sample order, using the ‘coniss’ clustering method from the R package ‘vegan’.

## Results

### Data Quality and Annotation

In total, 1077 OTUs were obtained from a 97% sequence similarity clustering; of these, 963 were classified as fungal and were considered for further analysis (Table 2 and suppl. S2). Of the 80 samples processed, 59 were considered of high quality (see suppl. S3 for OTU saturation curves). Sample-based OTU accumulation curves indicated that 96.6% of the expected total diversity was recovered by the sampling (suppl. S3 and S5). The 59 samples considered for further analysis had on average 95 ± 30 OTUs per sample after rarefaction (30,000 reads per sample).

**Table 2:** Overview on bioinformatics processing steps and taxonomic assignment

Assembled reads	3,542,865
OTUs (97% similarity)	1077
OTUs annotated to (accumulated percentage)	
Species	376 (34.9%)
Genus	209 (54.3%)
Family	30 (57.1%)
Order	162 (72.1%)
Class	55 (77.3%)
Phylum	90 (85.6%)
Kingdom (fungi)	41 (89.4%)
Unclassified	114 (100%)

### Temperature Data

**Fig. 2:**
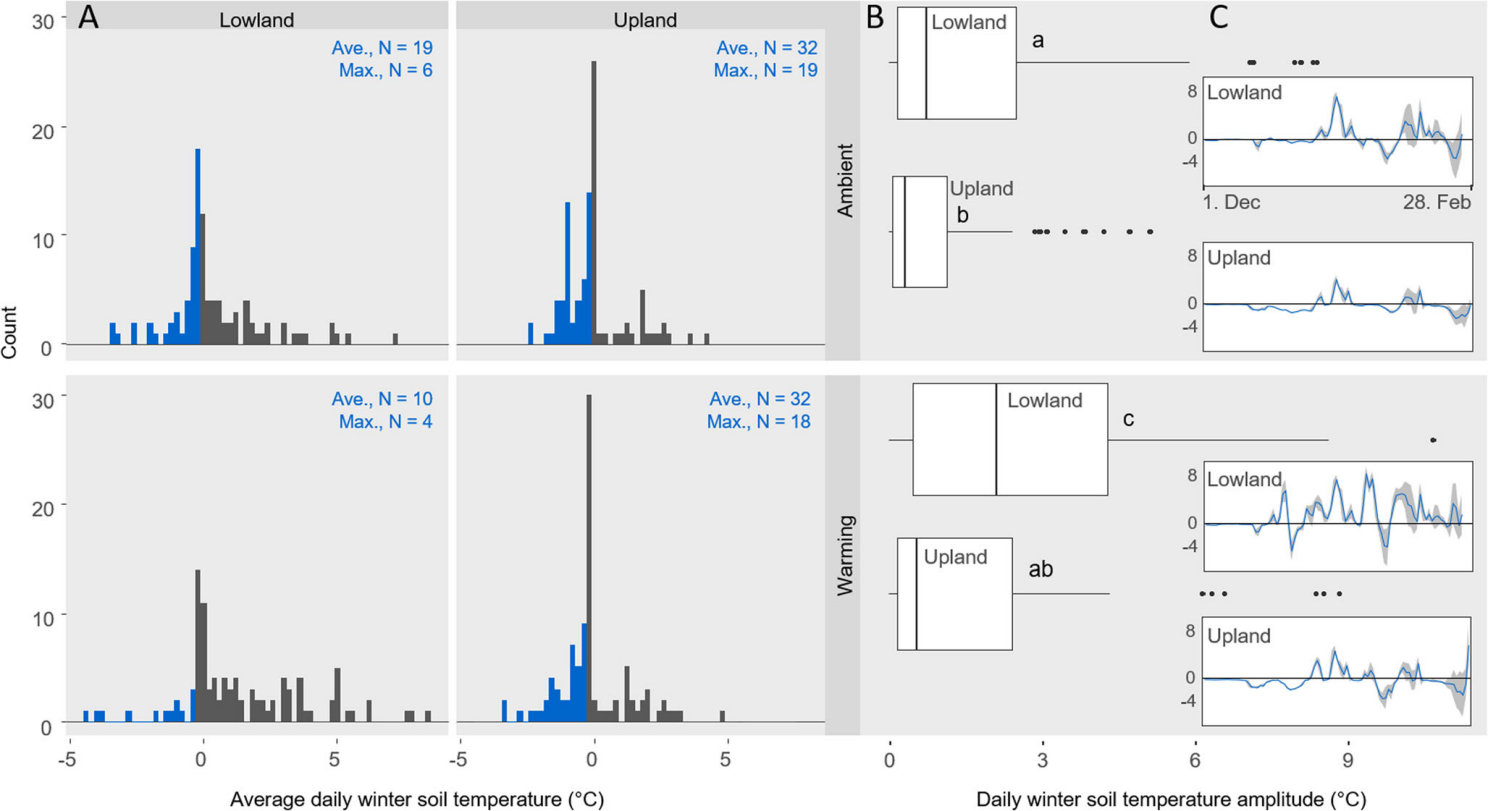
**a** Histogram of the average soil temperatures between Dec 1, 2010, and Feb 28, 2011 (bin width: 0.5 °C). The number of days (N) with average (Ave.) soil temperatures ≤ − 0.5 °C are indicated in blue and summarized in the upper right corner of each plot. Max. = the number of day with maximum soil temperatures ≤ − 0.5 °C. **b** Daily soil temperature fluctuation (difference between the maximum and minimum observed daily temperature). Boxes represent the first and third quartile, median (line) and outliers (dots) are indicated as are ANOVA results (*p* < 0.05; Tukey HSD groups: a–c). **c** Average daily soil temperature (blue line) and standard deviation (grey area) for the period. Data is a mean of two or four replicates from ambient and warming plots respectively

For the present study, frost events were considered a critical parameter for the mycobiome and when evaluating the temperature data with this focus we found that the number of days with average soil temperature below zero varied between sites, with 40.6% less days at the lowland site (19 vs. 32 days respectively, Fig. 2a). Likewise, the number of days with permanent sub-zero soil temperatures (i.e. maximum measured temperature within 24 h below − 0.5 °C) was more than tripled at the upland site compared to the lowland site (6 vs. 19 days respectively, Fig. 2a). The warming pulse treatment reduced these numbers to 10 and 4 days at the lowland site, while no reduction was seen at the upland site (Fig. 2a). The daily temperature fluctuation was significantly different between sites (*p* < 0.001) and treatments (*p* < 0.001, Fig. 2b), with the largest fluctuations seen at the lowland site (2.9 ± 3.1 °C, Fig. 2b and suppl. S5).

### Explanatory Power of Experimental Parameters for the Mycobiome

**Fig. 3:**
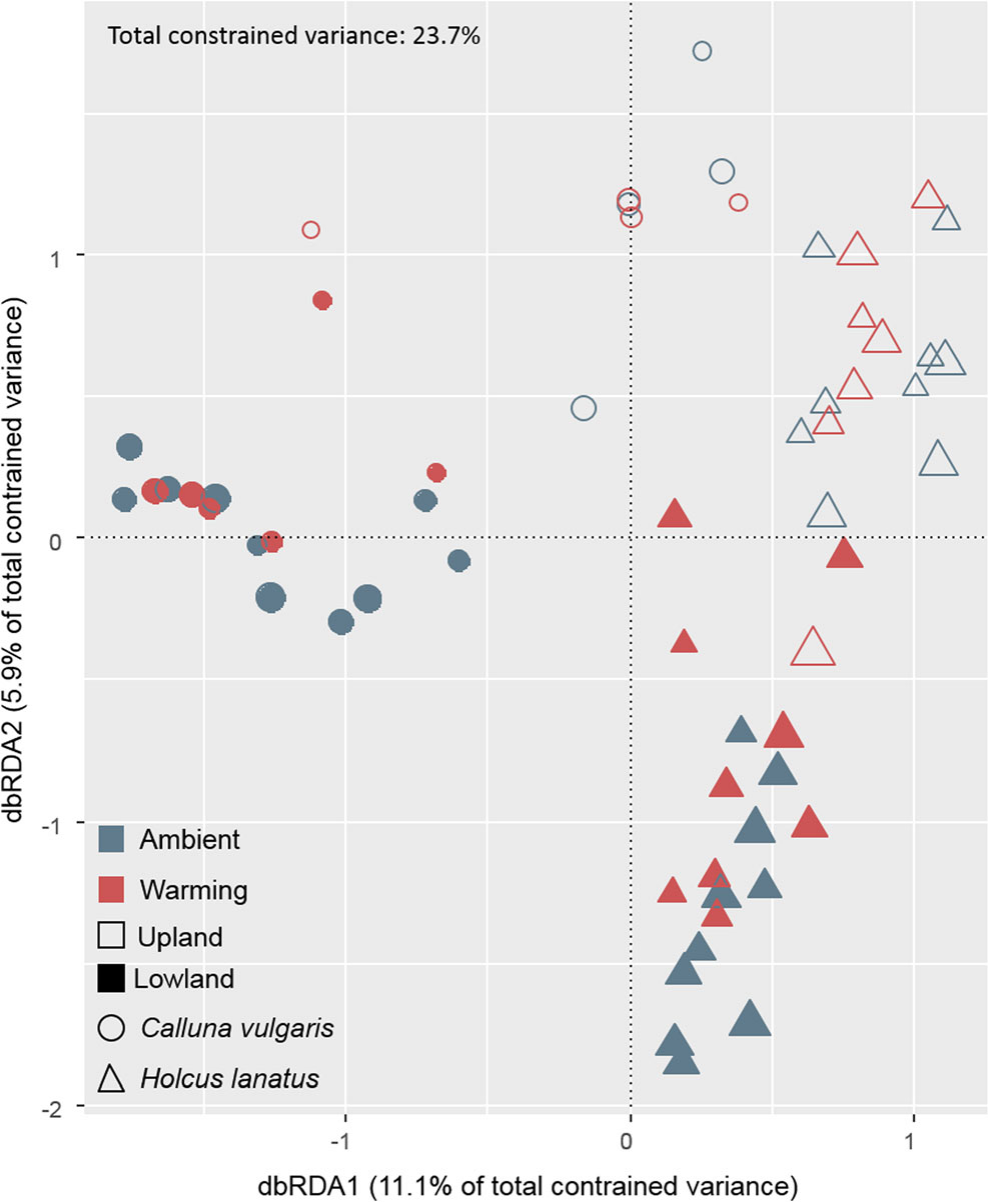
Distance based redundancy analysis (dbRDA) of the Bray-Curtis dissimilarity matrix of the fungal community (133 of 963 OTUs occurred in only one sample and were excluded for this plot). Symbols represent: *C. vulgaris* (circles), *H. lanatus* (triangle), upland (hollow), lowland (filled), ambient temperature (blue) and warming (red). Symbols are scaled to the number of OTUs in a given sample (ranging from 47 to 178 OTUs)

The total amount of mycobiome variance which could be explained by the assessed parameters was 23.7%, as revealed from distance-based redundancy analysis (dbRDA, Fig. 3 and Table 3). The first three dbRDA axes were found to significantly explain mycobiome variance (11.1%, 5.8% and 3.0% respectively, Table 3), with ‘plant species’ (*C. vulgaris*, *H. lanatus*), ‘site’ (upland, lowland) and the experimental ‘treatment’ (warming pulses) accounting for these axis (Table 3). All three parameters showed significant interaction effects indicating complex responses of the mycobiome (suppl. S5).

**Table 3:** Summary of distance-based redundancy analysis (dbRDA). First two rows show the ANOVA result for the axis and the proportion of constrained community variance explained by the axis (significant *p* values are in itals). Below are the biplot scores of the experimental variables (for each variable the highest axis scores are in italic)

	dbRDA1	bdRDA2	dbRDA3	dbRDA4	dbRDA5
Significance (*p*)	*0.001*	*0.001*	*0.001*	0.072	0.425
Explained variance (%)	11.1	5.8	3.0	2.1	1.5
Biplot scores for constraining variables					
Lowland	− 0.383	*− 0.531*	0.051	− 0.098	− 0.028
Upland	0.559	*0.774*	− 0.075	0.142	0.041
Ambient	− 0.019	− 0.108	*−0.672*	0.190	− 0.090
Warming	0.024	0.137	*0.853*	− 0.242	0.114
*Calluna vulgaris*	*− 0.853*	0.450	− 0.065	0.062	− 0.046
*Holcus lanatus*	*0.585*	− 0.309	0.045	− 0.043	0.032
Sampling date	0.142	0.092	0.270	0.529	*− 0.787*
No. days with soil frost	0.489	*0.680*	− 0.192	0.477	0.188

### Community Composition

**Fig. 4:**
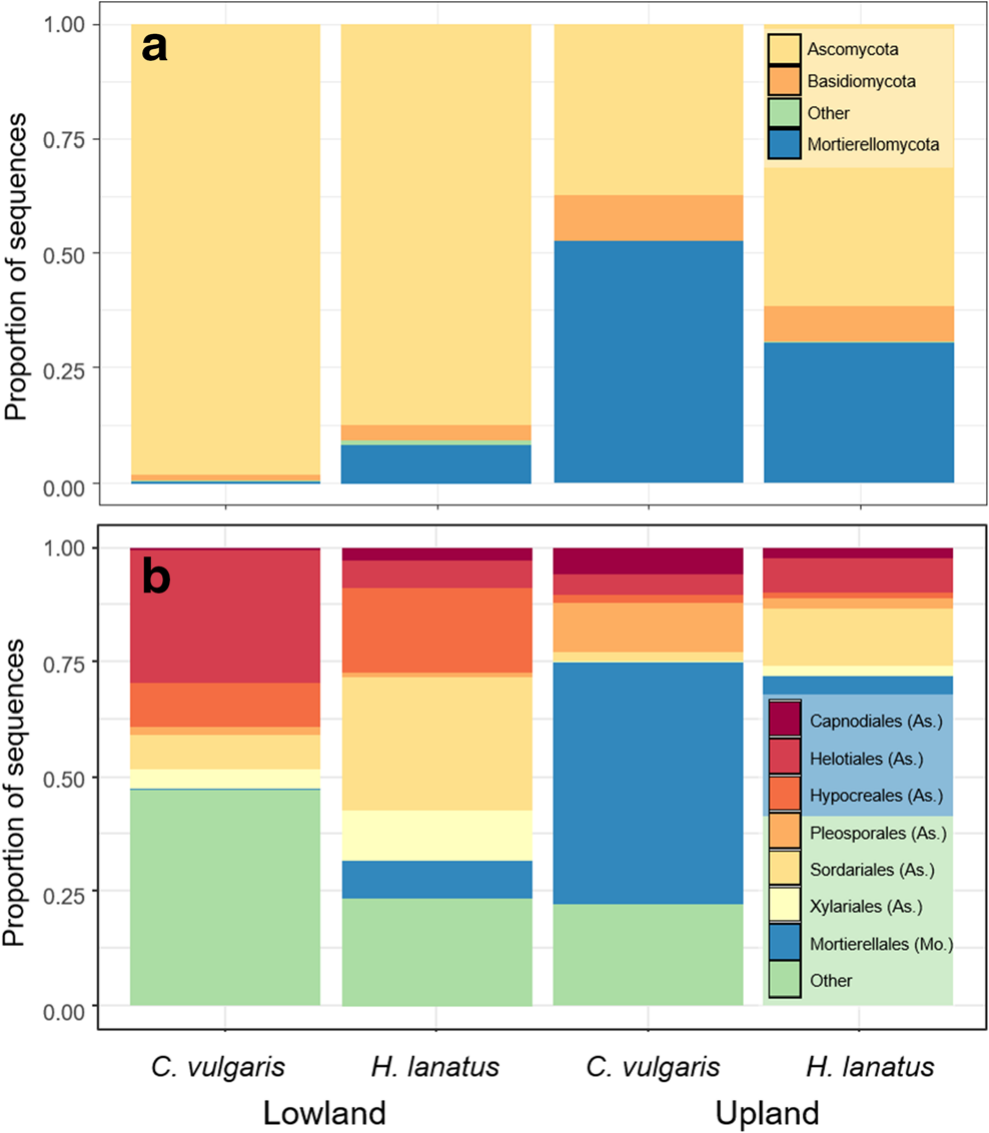
Relative abundance of the main phyla (**a**) and orders (**b**) for each site (upland and lowland) and plant species (*C. vulgaris* and *H. lanatus*). Taxa with < 2% relative abundance were summarized as ‘other’, for **b** also unclassified OTUs of Ascomycota and Dothideomycetes were placed under ‘others’. As. = Ascomucota, Mor. = Mortierellomycota

The mycobiome comprised mainly of taxa assigned to the Ascomycota (76.0 ± 30.7% of total relative abundance), among classified OTUs Sordariomycetes (15.2%) and Helotiales (11.5%) were dominant (Fig. 4a–b). Second most dominant phylum was the Mortierellomycota (18.4 ± 30.2%; all belonging to Mortierellales), whereas only a smaller fraction belonged to the Basidiomycota (5.3 ± 7.4%; Agaricomycetes, 2.8% and Tremellomycetes, 1.7%, Fig. 4a–b). A significantly (*p* = 0.041) larger fungal diversity was observed for *H. lanatus* compared to *C. vulgaris* mesocosms (Table 4; for taxonomic composition of individual samples, see suppl. S6).

**Table 4:** Summary of OTU richness, main phyla (Asco-. Basidio- and Mortierellomycota) and functional classes recovered from the two sites (Up: upland, Low: lowland), plant species (Cv: *Calluna vulgaris*, Hl: *Holcus lanatus*), treatment (A, ambient; W, warming) and sampling time (Sp, spring; Au, autumn). Values are given as mean ± SD for the number of OTUs (1st line) and their relative abundance (2nd line) per sample. Data was normalized to 30,000 reads per sample prior to analysis. Significant differences within the four categories are indicated with *p < 0.05, **p < 0.01, ***p < 0.001 (Kruskal-Wallis test)

	Site (up/low)	Plant species (Cv/Hl)	Treatment (A/W)	Sampling time (Sp/Au)
Investigated samples	24/35	24/35	33/26	26/33
OTUs per sample	91 ± 35/97 ± 27	85 ± 27/101 ± 31*	100 ± 30/88 ± 32	87 ± 24/103 ± 33*
Ascomycota	58.7 ± 24.9/75.7 ± 23.6** (53.8 ± 33.4%/91.2 ± 16.5%)***	62.2 ± 26.7/73.3 ± 23.8 (76.3 ± 35.5%/75.8 ± 27.5%)	75 ± 25.6/60.8 ± 23.3* (80.1 ± 31.2%/70.7 ± 29.8%)	63.4 ± 22.8/73 ± 26.8 (83.3 ± 27.8%/70.2 ± 32%)
Basidiomycota	8.9 ± 5/7.4 ± 3.9 (8.5 ± 9.3%/3.1 ± 4.9%)**	7 ± 4.6/8.8 ± 4.2 (5.2 ± 7.7%/5.4 ± 7.4%)	7.8 ± 4.1/8.3 ± 4.8 (4.5 ± 7.2%/6.2 ± 7.7%)	8.2 ± 3.9/7.9 ± 4.8 (4.8 ± 7.1%/5.6 ± 7.8%)
Mortierellomycota	19.7 ± 10.9/7.9 ± 6.5*** (37.6 ± 36.8%/5.2 ± 14%)***	11 ± 9.7/13.9 ± 10.6 (18.6 ± 35.7%/18.2 ± 26.3%)	11.5 ± 9.1/14.3 ± 11.6 (15 ± 31.5%/22.7 ± 28.4%)	8 ± 9.3/16.5 ± 9.6*** (11.5 ± 26.6%/23.8 ± 32.1%)***
Wood Saprotroph (5.2% of all fungal OTUs)	3 ± 4/5 ± 5 (4 ± 13.4%/1.8 ± 4.5%)	4 ± 6/4 ± 4 (2.6 ± 6.6%/2.8 ± 10.7%)	5 ± 5/3 ± 3 (2.4 ± 5.7%/3.1 ± 12.4%)	4 ± 5/4 ± 5 (1.6 ± 4.6%/3.6 ± 11.6%)
Saprotroph-Symbiotroph (11.4% of all fungal OTUs)	21 ± 11/9 ± 7*** (41.3 ± 35.4%/5.7 ± 14.4%)***	11 ± 10/16 ± 11 (18.5 ± 35.6%/21.3 ± 27%)	13 ± 9/15 ± 12(17.9 ± 32.1%/23.1 ± 28.7%)	9 ± 9/18 ± 10*** (12.6 ± 26.7%/26.1 ± 32.4%)*
Saprotroph (22.5% of all fungal OTUs)	22 ± 9/17 ± 8*** (21.2 ± 21.2%/17.1 ± 22.2%)	14 ± 6/23 ± 9*** (8.4 ± 10%/25.9 ± 24.7%)**	21 ± 8/16 ± 9 (21.7 ± 20.9%/15.1 ± 22.6%)*	16 ± 8/21 ± 9*** (20.3 ± 24%/17.6 ± 20%)
Ericoid Mycorrhizal (3.3% of all fungal OTUs)	1 ± 2/6 ± 6*** (0.0 ± 0.0%/12 ± 23.8%)***	8 ± 6/1 ± 1*** (17.2 ± 27.2%/0.2 ± 1%)***	4 ± 6/4 ± 5 (7.1 ± 17%/7.1 ± 21.9%)	5 ± 6/3 ± 5 (11.1 ± 25.5%/3.9 ± 11.5%)
Ectomycorrhizal (2.4% of all fungal OTUs)	1 ± 1/3 ± 2*** (1.2 ± 3.9%/1.0 ± 2.6%)	3 ± 3/1 ± 2*** (1.1 ± 3.3%/1.1 ± 3.1%)	3 ± 2/1 ± 1*** (1.2 ± 3.2%/0.9 ± 3.2%)	3 ± 2/2 ± 2 (0.9 ± 2.9%/1.2 ± 3.4%)
Arbuskular Mycorrhizal (1.1% of all fungal OTUs)	0.3 ± 0.7/0.5 ± 0.9 (0.1 ± 0.4%/0 ± 0.2%)	0.4 ± 0.8/0.3 ± 0.8 (0 ± 0%/0.1 ± 0.3%)	0.3 ± 0.8/0.4 ± 0.9 (0.1 ± 0.3%/0.1 ± 0.2%)	0.3 ± 0.7/0.4 ± 0.9 (0.1 ± 0.2%/0.1 ± 0.3%)

Of the 922 OTUs assigned to minimum phyla-level, 651 (representing 69.0% of the total abundance) could be assigned to a functional group using the FUNguild classifier (Table 5). Of these, ca. half (326 OTUs representing 29.1% of the total abundance) was assigned with a high confidence score (‘highly probable’ or ‘probable’, Table 5). A shift in taxonomic composition was seen between the upland and lowland sites, which was also reflected in the functional profile of the mycobiome, where saprotrophic fungi had an increased richness and the group saprotrophic-symbiotrophs (including Mortierellales) a large increase in relative abundance at the upland site (41.3 ± 35.4% vs. 5.7 ± 14.4% at the upland and lowland site respectively, Table 4). On the contrary, mycorrhizal fungi both assigned to ecto- and ericoid mycorrhiza were strongly reduced in richness at the upland site and for the latter its occurrence at the upland site completely diminished (12 ± 23.8% to < 0.0% at the lowland and upland sites respectively, Table 4). Arbuscular mycorrhiza showed no response and had in general a very low abundance (< 1%, Table 4).

**Table 5:** Summary of FUNguild classification. Functional groups with a total abundance < 0.01 across confidence score is summarized under ‘other’, this count: ‘Arbuscular mycorrhizal’, ‘Fungal parasite-litter saprotroph’, ‘pathotroph-saprotroph’ and ‘symbiotroph’

Classification	Confidence	Abundance	Richness
Ectomycorrhizal	Highly probable	0.1%	7
Ectomycorrhizal	Probable	1.0%	13
Ectomycorrhizal	Possible	0.02%	3
Ericoid mycorrhizal	Probable	6.4%	29
Ericoid mycorrhizal	Possible	0.7%	3
Pathotroph-symbiotroph	Probable	0.6%	22
Pathotroph-symbiotroph	Possible	10.3%	65
Plant pathogen	Highly probable	0.0002%	1
Plant pathogen	Probable	7.0%	77
Saprotroph	Highly probable	0.3%	10
Saprotroph	Probable	11.9%	135
Saprotroph	Possible	6.6%	72
Saprotroph-symbiotroph	Highly probable	0.001%	1
Saprotroph-symbiotroph	Probable	0.03%	4
Saprotroph-symbiotroph	Possible	20.2%	105
Wood Saprotroph	Highly probable	0.2%	9
Wood saprotroph	Probable	1.6%	18
Wood saprotroph	Possible	0.9%	23
Other	NA	1.3%	54
Sum assigned		69.0%	651
Not assigned	–	31.0%	271

**Fig. 5:**
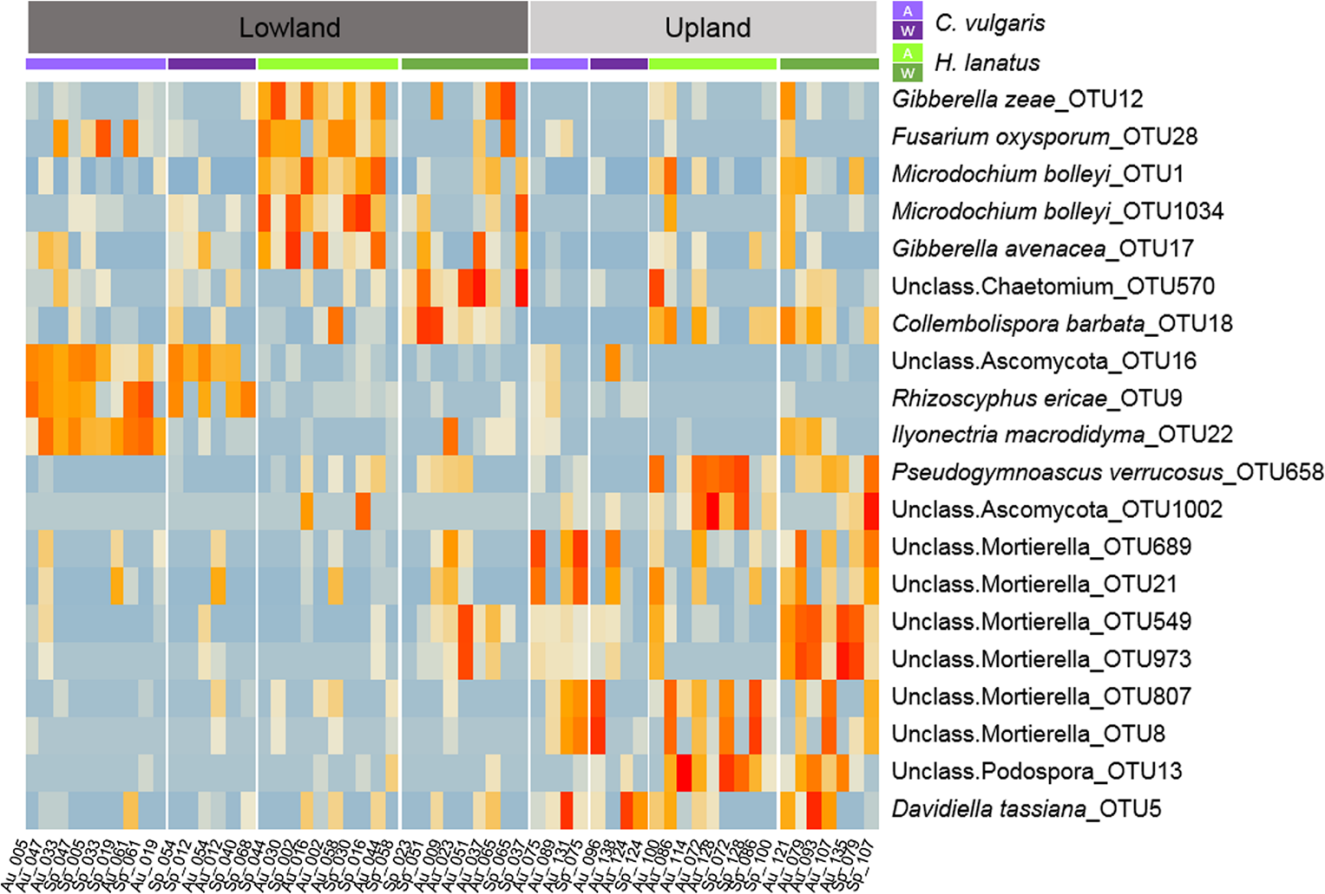
Heatmap of the ten most frequent and abundant OTUs from each site (upland and lowland), representing 43.1% of the total community (rarefied). Data was normalized to 30,000 reads per sample prior to analysis. Red colours indicate relatively high abundance; blue colours indicate relatively low abundance for a given OTU across samples. OTUs with similar distribution pattern across samples are clustered. Upper colour bar represents the sample sorting by plant species *C. vulgaris* (purple) and *H. lanatus* (green) and treatment: ambient (A, light colour) and warming (W, dark colour). Sample ID is given at the bottom (Au: Autumn sample, Sp: Spring sample). Taxonomic classification and OTU ID are given at the right side

The mycobiome showed clear plant-specific ‘signatures’ at the lowland site, an effect largely driven by symbiont species (Fig. 5), whereas at the upland site plant-specific community patterns could no longer be identified. Here the communities were dominated by OTUs assigned to the genus *Mortierella*, a pattern consistent when summarized at genera level (suppl. S7).

For the mycobiome associated with *C. vulgaris*, a pronounced difference between the two sites occurred in the abundance of the ericoid mycorrhizal partner *Rhizoscyphus* (syn: *Hymenoscyphus* and *Pezizella*) *ericae* [47, 48] which was highly dominant at the lowland site but almost completely absent at the upland site. Likewise, in the mycobiome of *H. lanatus* though no arbuscular mycorrhizal fungi was among the dominant taxa, both *Microdochium bolleyi* and *Chaetomium* sp. were dominant at the lowland site but highly reduced at the upland site. Both these taxa (*M. bolleyi* and *Chaetomium* sp.) are known as beneficial root associated fungi involved in pathogen protection for the grass family *Poaceae* and other herbaceous plant species [49–53]. A number of potential plant pathogens were also among the dominant fungi associated with both *H. lanatus* (*Fusarium oxysporum*, *Gibberella zeae* and *G. avenacea*; the latter two both known to cause epidemics of Fusarium head blight in wheat crops as well as wild grasses [54]) and *C. vulgaris* (*Ilyonectria macrodidyma*; a root pathotroph of woody plants [55, 56]) at the lowland site, but had likewise a reduced abundance at the upland site.

## Discussion

### Local Winter Climate Conditions Had Profound Effects on the Mycobiome

Surprisingly, the plant-specific mycobiome patterns were strongly diminished at the upland site for both ambient and warming treated mesocosms (Fig. 5). Since no real difference in the community was observed between the first and second sampling campaign (i.e. between early spring and late autumn; Fig. 5 and suppl. S5), the changes in mycobiome at the upland site seem related to winter climate conditions. This was in accordance with the pattern observed at the lowland site, where the experimental warming treatment led to a community shift comparable to that observed between the control treatment at the lowland and the upland site (Figs. 3 and 5). In line with the predictions for relatively warm and cold temperate sites [4–6], the warming treatment reduced the number of days with soil frost at the lowland site and increased the daily soil temperature fluctuations (Fig. 2b and Schuerings et al. 2014a [18]), while at the upland site only a small (non-significant) increase in temperature fluctuation was seen and the number of days with soil frost remained unchanged (and much higher than at the lowland site, 32 days; Fig. 2a–b). These different consequences of the experimental treatment for the soil temperature at the two sites likely explained why the experimental winter warming alone was only found to explain a minor proportion (3.0%) of the mycobiome variance (dbRDA3, Table 3). We suspect warming treatment effects at the upland site were masked by the already much altered (i.e. colder and frost-effected) climate at this site. Urakawa et al. (2014) [57] conducted a transplant experiment comparable to ours with in situ incubations of ten different soils from temperate forest sites, transplanted to sites experiencing either non-frozen and frost-effected winters in Japan. The authors found microbial processes (nitrogen mineralisation, nitrification and denitrification) highly affected by winter climate both during winter and in the following growing season. In addition, the authors reported the magnitude and frequency of freeze-thaw cycles as important explanatory parameter of their results, and suggested that soils experiencing high intensity of freeze-thaw cycles hosted microbial communities with higher tolerance to such. Other authors have found changes in nitrogen dynamics post-freezing to be linked to root mortality and changes in plant community in temperate grassland and broadleaf forest [10, 21]. In the present study, the plant community composition was controlled and increased root growth was reported for both species under the experimental conditions, why we expect changes in N mineralisation was mainly driven by the microbial community.

The mesocosm design allowed us to establish and contain systems in a close-to-natural fashion, making the results more relatable to natural ecosystems compared to laboratory experiments. However, being that it is an only partly controlled design, several environmental parameters will differ. Thus, it cannot be ruled out that other factors besides temperature contributed to the observed changes in the mycobiome between the upland and lowland site. For example, the mean winter precipitation is generally higher at the upland site [18], which was also the case during the winter of the experimental year 2010/2011, where total precipitation was 400 mm and 314 mm from November to March at the upland and lowland site respectively (daily precipitation did not significantly differ between the two sites, suppl. S1). The mesocosms were designed as zero tension containers (i.e. always drained, see the ‘Experimental Design’ section); however, precipitation and other environmental parameters may still have contributed to the observed community change.

### A New Community Balance at the Upland Site

The plant symbiont species were diminished and instead, saprotrophic fungi were dominant at the upland site, with a large dominance of members of the genus *Mortierella* but also *Pseudogymnoascus verrucosus*, the latter a psychrotolerant soil saprotroph [58]. Coinciding, a high extracellular enzymatic activity and a significant increase in plant-available nitrogen (nitrate and ammonium) was previously reported for the upland site (Table 1 and Schuerings et al. 2014a, 2014b [15, 18]). These results suggest that easily accessible substrates were available at the upland site during the winter 2010/2011. A possible source of nitrogen rich substrate may have been microbial necromass, resulting from the strong community turn-over which had taken place at the upland site. We suspect that the soil at the upland site experienced a microbial die-off upon transplantation to the upland site potentially caused by the colder environmental conditions at this site (increased degree of soil freezing) resulting in the observed taxonomic alterations and the dead microbial biomass acting as a source of easily degradable substrates [59]. This interpretation is in line with studies showing soil freezing as a strong modifier of microbial community composition often resulting in an initial microbial death upon freezing and a compositional shift toward frost tolerant species [60–62].

The present study only targeted the fungi and thus nothing is known about the dynamics of the remaining members of the microbiota in the soils. The degree and frequency of soil freezing have been reported to explain microbial (bacteria, fungi and amoeba) community composition in natural (alpine) soil ecosystems [63–65]. In our study, a prominent example of a likely frost-induced change in abundance was seen for *Fusarium oxysporum*, a species found to be highly sensitive to freezing under laboratory conditions [66] and which was among the dominant taxa at the lowland site but was strongly reduced at the upland site (Fig. 5). An altered microbial community structure will undoubtedly influence the mineralisation and availability of nutrients in the soil. As such, a shift in the relative dominance between e.g. bacteria and fungi could also have resulted in the increased nitrogen availability reported from the upland site and warming treatment [62, 67]. Kuffner et al. (2012) [68] found no changes in richness and only minor changes in taxonomic composition of bacteria across season in a warming manipulation study conducted on subalpine forest soils in the Austrian Alps. Those results are in accordance with other studies showing that bacteria are generally more freeze-thaw tolerant than both fungi and archaea [13, 60, 68]. Also, protists and other less studied microbial organisms will likely have species-specific responses to the altered environmental condition e.g. some amoebae (naked amoebae) have been reported as highly frost tolerant [69] while others (myxamoeba) were shown to be highly sensitive to freezing and even more so when these occur rapidly, leaving no time for encystment and/or other coping mechanisms [19]. Likewise, the speed of freezing has also been shown to affect the survival rate of bacteria [70] and finally, Tibbett et al. (2002) [71] found the recovery rate of ectomycorrhizal fungi after a freezing event partly explained by the temperature prior to freezing (2 °C or 22 °C), suggesting that a wider temperature amplitude (as seen for the warming treatment at the lowland site) can result in a decreased frost-tolerance for some fungi. In the case of an increase in microbial necromass at the upland site, this might have acted as an intermediate plant growth driver (which must be considered transient). Under natural conditions (non-monoculture conditions), a shift in the plant community can be expected upon shifts in the fungal community, although the plant response might not follow immediately [72, 73]. Our results suggest that such decoupled response between fungi and plants was undertaking.

### Reduced Abundance of Fungal Symbionts Coinciding with Positive Plant Growth Responses

A positive growth response was reported for *C. vulgaris* at the upland site, suggesting that the lack of symbiont *R. ericae* did not reduce the fitness of the host plant. Both *C. vulgaris* and *H. lanatus* are common plant species in temperate and sub-arctic ecosystems and should have no problem prevailing at the mountainous upland site. Although studies have shown that both arbuscular and ectomycorrhizal fungi can survive hard frost events [74], ericoid mycorrhizal associations of Ericaceae plants (including *C. vulgaris*) have been reported to decline with increased latitude and altitude [75, 76], and Vohník and Albrechtová (2011) [77] reported a shift in abundance between ericoid mycorrhizal and the so-called dark-septate endophyte fungi associated with the plant genus *Rhododendron* (Ericaceae) with increased altitude and latitude across Europe. Common for these studies, however, is that the shifts are generally seen at considerably colder regions than our upland site (i.e. alpine and sub-arctic). Thus, the reduction in abundance of the ericoid fungi *R. ericae* at the upland site might be related to other parameters than temperature *per se*. The host plant *C. vulgaris* might be capable of an efficient regulation of its mycorrhizal associations under favourable environmental condition (such as N availability) allowing the plant to put its carbon resources in to growth rather than supplying the mycosymbiont. A reduction in mycorrhizal associations under high nutrient availability has previously been reported for ecto- and arbuscular mycorrhizal associations [78–82]. Experiments on the effect of ericoid mycorrhizal partners on plant growth under nutrient enriched condition have to our knowledge not yet been carried out.

In addition, both plant species experienced a release from pathogens at the upland site (from *I. macrodidyma* and *F. oxysporum*/*G. zeae*/*G. avenacea* for *C. vulgaris* and *H. lanatus* respectively) which might contribute to explaining the positive growth response previously reported for both plant species at this site [15, 18] (Table 1). Likewise, *I. macrodidyma* was reduced under the warming-pulse treatment at the lowland site, and also here a positive growth response for *C. vulgaris* was reported [18] (above-ground biomass and root length; Table 1). Although the warming-pulses at the lowland site reduced the soils exposure to frost, a compositional change among the dominant members of the mycobiome, comparable to that seen for the upland site, was observed at this site (Fig. 3). This was especially true for the mycobiome of *H. lanatus,* where the abundance of the plant-beneficial fungi *M. bolleyi* decreased under the warming-pulse treatment (Fig. 5). Subsequently, a lower biomass of *H. lanatus* was reported from the following growing season. Both examples point to direct effects of the mycobiome on plant growth (Table 1).

## Conclusion

Increased temperature fluctuations and extreme climatic events are predicted to become more frequent with climate change [83]. Based on our results, this can have potentially far reaching consequences for soil mycobiomes and subsequently plant productivity. Furthermore, our results indicate that legacy effects after such ‘extreme’ events can be expected to remain in the ecosystem across seasons (i.e. no observed difference between the first and second sampling).

In summary, our results suggest that years with weather conditions outside the common climatic range for the given community (here seen at the upland site and under warming condition at the lowland site) and winter climate (not summer) have the potential to drive drastic changes in the mycobiome, with ecosystem-scale consequences for mineralisation and plant growth. The results also suggest that the mycobiome responds similarly to a combination of cold temperature stressors i.e. fluctuations (including number of freeze-thaw cycles), rate of freezing, absolute minimum temperature and duration of freezing. More research is needed to identify specific thresholds for these stressors as well as microbial community structure resilience.

## Supplementary Information

Supplementary Material 1 Precipitation data. (XLSX 79 kb)

Supplementary Material 2 OTU tables. (CSV 163 kb)

Supplementary Material 3 DNA- and sample-based OTU accumulation curves. (PDF 253 kb)

Supplementary Material 4 Analysis script. (R 44 kb)

Supplementary Material 5 Raw results incl. parameter interactions. (TXT 37 kb)

Supplementary Material 6 Bar chart showing individual sample variance. (PNG 240 kb)

Supplementary Material 7 Heatmap of summarized community (genus level). (PDF 132 kb)

## References

[1] Matzner E, Borken W (2008). Do freeze-thaw events enhance C and N losses from soils of different ecosystems? A review. Eur J Soil Sci.

[2] Kreyling J (2010). Winter climate change: a critical factor for temperate vegetation performance. Ecology.

[3] Ladwig LM, Ratajczak ZR, Ocheltree TW, Hafich KA, Churchill AC, Frey SJK, Fuss CB, Kazanski CE, Muñoz JD, Petrie MD, Reinmann AB, Smith JG (2016). Beyond arctic and alpine: the influence of winter climate on temperate ecosystems. Ecology.

[4] Groffman PM, Driscoll CT, Fahey TJ, Hardy JP, Fitzhugh RD, Tierney GL (2001). Colder soils in a warmer world: a snow manipulation study in a northern hardwood forest ecosystem. Biogeochemistry.

[5] Kreyling J, Henry HAL (2011). Vanishing winters in Germany: soil frost dynamics and snow cover trends, and ecological implications. Clim Res.

[6] Christensen J, Hewitson B, Busuioc A, Solomon S, Qin D, Manning M, Chen Z, Marquis M (2007). Regional climate projections. Climate change 2007: the physical science basis. Contribution of working group I to the fourth assessment report of the intergovernmental panel on climate change.

[7] Kodra E, Steinhaeuser K, Ganguly AR (2011). Persisting cold extremes under 21^st^ -century warming scenarios. Geophys Res Lett.

[8] Henry HAL (2008). Climate change and soil freezing dynamics: historical trends and projected changes. Clim Chang.

[9] Brown PJ, DeGaetano AT (2011) A paradox of cooling winter soil surface temperatures in a warming northeastern United States. Agric For Meteorol 151:947–956. 10.1016/j.agrformet.2011.02.014

[10] Tierney GL, Fahey TJ, Groffman PM (2003). Environmental control of fine root dynamics in a northern hardwood forest. Glob Chang Biol.

[11] Oztas T, Fayetorbay F (2003). Effect of freezing and thawing processes on soil aggregate stability. Catena.

[12] Jefferies RL, Walker NA, Edwards KA, Dainty J (2010). Is the decline of soil microbial biomass in late winter coupled to changes in the physical state of cold soils?. Soil Biol Biochem.

[13] Schimel J, Balser T, Wallenstein M (2007). Microbial stress-response physiology and its implications for ecosystem function. Ecology.

[14] Kreyling J (2019) The ecological importance of winter in temperate, boreal, and arctic ecosystems in times of climate change. Prog Bot [in press]. 10.1007/124_2019_35

[15] Schuerings J, Jentsch A, Hammerl V (2014). Increased winter soil temperature variability enhances nitrogen cycling and soil biotic activity in temperate heathland and grassland mesocosms. Biogeosciences.

[16] Sasse J, Martinoia E, Northen T (2018). Feed your friends: do plant exudates shape the root microbiome?. Trends Plant Sci.

[17] Berg G, Rybakova D, Grube M, Köberl M (2016). The plant microbiome explored: implications for experimental botany. J Exp Bot.

[18] Schuerings J, Jentsch A, Walter J, Kreyling J (2014). Winter warming pulses differently affect plant performance in temperate heathland and grassland communities. Ecol Res.

[19] Shchepin O, Novozhilov Y, Schnittler M (2014). Protistology Nivicolous myxomycetes in agar culture: some results and open problems. Potistology.

[20] Brooks PD, Williams MW, Schmidt SK (1998). Inorganic nitrogen and microbial biomass dynamics before and during spring snowmelt. Biogeochemistry.

[21] Joseph G, Henry HAL (2008). Soil nitrogen leaching losses in response to freeze-thaw cycles and pulsed warming in a temperate old field. Soil Biol Biochem.

[22] Robinson CH (2001). Cold adaptation in Arctic and Antarctic fungi. New Phytol.

[23] Klironomos JN, Hart MM, Gurney JE, Moutoglis P (2001). Interspecific differences in the tolerance of arbuscular mycorrhizal fungi to freezing and drying. Can J Bot.

[24] Lekberg Y, Koide RT (2008). Effect of soil moisture and temperature during fallow on survival of contrasting isolates of arbuscular mycorrhizal fungi. Botany.

[25] Turan M, Yildirim E, Kitir N, Zaidi A, Khan M (2017). Beneficial role of plant growth-promoting bacteria in vegetable production under abiotic stress. Microbial strategies for vegetable production.

[26] Askari-Khorasgani O, Hatterman-Valenti H, Flores Pardo FB, Pessarakli M (2019). Plant and symbiont metabolic regulation and biostimulants application improve symbiotic performance and cold acclimation. J Plant Nutr.

[27] Wang C, Wang C, Gao YL, Wang YP, Guo JH (2016). A consortium of three plant growth-promoting rhizobacterium strains acclimates *Lycopersicon esculentum* and confers a better tolerance to chilling stress. J Plant Growth Regul.

[28] Chen S, Jin W, Liu A, Zhang S, Liu D, Wang F, Lin X, He C (2013). Arbuscular mycorrhizal fungi (AMF) increase growth and secondary metabolism in cucumber subjected to low temperature stress. Sci Hortic.

[29] Theocharis A, Bordiec S, Fernandez O, Paquis S, Dhondt-Cordelier S, Baillieul F, Clément C, Barka EA (2012). *Burkholderia phytofirmans* PsJN primes *Vitis vinifera* L. and confers a better tolerance to low nonfreezing temperatures. Mol Plant-Microbe Interact.

[30] Kreyling J, Peršoh D, Werner S, Benzenberg M, Wöllecke J (2012). Short-term impacts of soil freeze-thaw cycles on roots and root-associated fungi of *Holcus lanatus* and *Calluna vulgaris*. Plant Soil.

[31] Gardes M, Bruns TD (1993). ITS primers with enhanced specificity for basidiomycetes - application to the identification of mycorrhizae and rusts. Mol Ecol.

[32] White TJ, Brunns T, Lee S, Taylor J (1990) Amplification and direct sequencing of fungal ribosomal TNA genes for phylogenetics

[33] Peršoh D, Stolle N, Brachmann A, Begerow D, Rambold G (2018). Fungal guilds are evenly distributed along a vertical spruce forest soil profile while individual fungi show pronounced niche partitioning. Mycol Prog.

[34] Caporaso JG, Kuczynski J, Stombaugh J, Bittinger K, Bushman FD, Costello EK, Fierer N, Peña AG, Goodrich JK, Gordon JI, Huttley GA, Kelley ST, Knights D, Koenig JE, Ley RE, Lozupone CA, McDonald D, Muegge BD, Pirrung M, Reeder J, Sevinsky JR, Turnbaugh PJ, Walters WA, Widmann J, Yatsunenko T, Zaneveld J, Knight R (2010). QIIME allows analysis of high- throughput community sequencing data. Nat Methods.

[35] Guerreiro MA, Brachmann A, Begerow D, Peršoh D (2018). Transient leaf endophytes are the most active fungi in 1-year-old beech leaf litter. Fungal Divers.

[36] Röhl O, Graupner N, Peršoh D, Kemler M, Mittelbach M, Boenigk J, Begerow D (2018). Flooding duration affects the structure of terrestrial and aquatic microbial eukaryotic communities. Microb Ecol.

[37] Huang Y, Niu B, Gao Y, Fu L, Li W (2010). CD-HIT Suite: a web server for clustering and comparing biological sequences. Bioinformatics.

[38] Röhl O, Peršoh D, Mittelbach M, Elbrecht V, Brachmann A, Nuy J, Boenigk J, Leese F, Begerow D (2017). Distinct sensitivity of fungal freshwater guilds to water quality. Mycol Prog.

[39] Kõljalg U, Nilsson RH, Abarenkov K, Tedersoo L, Taylor AFS, Bahram M, Bates ST, Bruns TD, Bengtsson-Palme J, Callaghan TM, Douglas B, Drenkhan T, Eberhardt U, Dueñas M, Grebenc T, Griffith GW, Hartmann M, Kirk PM, Kohout P, Larsson E, Lindahl BD, Lücking R, Martín MP, Matheny PB, Nguyen NH, Niskanen T, Oja J, Peay KG, Peintner U, Peterson M, Põldmaa K, Saag L, Saar I, Schüßler A, Scott JA, Senés C, Smith ME, Suija A, Taylor DL, Telleria MT, Weiss M, Larsson KH (2013). Towards a unified paradigm for sequence-based identification of fungi. Mol Ecol.

[40] Spatafora JW, Chang Y, Benny GL, Lazarus K, Smith ME, Berbee ML, Bonito G, Corradi N, Grigoriev I, Gryganskyi A, James TY, O’Donnell K, Roberson RW, Taylor TN, Uehling J, Vilgalys R, White MM, Stajich JE (2016). A phylum-level phylogenetic classification of zygomycete fungi based on genome-scale data. Mycologia.

[41] Chao A, Jost L (2012). Coverage-based rarefaction and extrapolation: standardizing samples by completeness rather than size. Ecology.

[42] Nguyen NH, Song Z, Bates ST, Branco S, Tedersoo L, Menke J, Schilling JS, Kennedy PG (2016). FUNGuild: an open annotation tool for parsing fungal community datasets by ecological guild. Fungal Ecol.

[43] R Core Team (2019) R: a language and environment for statistical computing. R Found Stat Comput

[44] Oksanen J, Guillaume BF, Friendly M (2019). Vegan: community ecology package. R package version.

[45] Chao A, Colwell RK (2014) Rarefaction and extrapolation with Hill numbers: a framework for sampling and estimation in species diversity studies. Ecol Monogr 84:45–67. 10.1890/13-0133.1

[46] Anderson MJ (2001). A new method for non parametric multivariate analysis of variance. Austral Ecol.

[47] Pearson V, Read DJ (1975). The physiology of the mycorrhizal endophyte of *Calluna vulgaris*. Trans Br Mycol Soc.

[48] Sokolovski SG, Meharg AA, Maathuis FJM (2002). *Calluna vulgaris* root cells show increased capacity for amino acid uptake when colonized with the mycorrhizal fungus *Hymenoscyphus ericae*. New Phytol.

[49] Zhang W, Krohn K, Draeger S, Schulz B (2008). Bioactive isocoumarins isolated from the endophytic fungus *Microdochium bolleyi*. J Nat Prod.

[50] Douglas LI, Deacon JW (1994). Strain variation in tolerance of water stress by *Idriella* (*Microdochium*) *bolleyi*, a biocontrol agent of cereal root and stem base pathogens. Biocontrol Sci Tech.

[51] Hung PM, Wattanachai P, Kasem S, Poeaim S (2015). Efficacy of *Chaetomium* species as biological control agents against *Phytophthora nicotianae* root rot in citrus. Mycobiology.

[52] Soytong K, Kanokmedhakul S, Kukongviriyapa V, Isobe M (2001). Application of *Chaetomium* species spectrum biological review article fungicide. Fungal Divers.

[53] Park JH, Gyung JC, Kyoung SJ (2005). Antifungal activity against plant pathogenic fungi of chaetoviridins isolated from *Chaetomium globosum*. FEMS Microbiol Lett.

[54] Inch S, Gilbert J (2003). The incidence of Fusarium species recovered from inflorescences of wild grasses in southern Manitoba. Can J Plant Pathol.

[55] Úrbez-Torres JR, Peduto F, Gubler WD (2012). First report of *Ilyonectria macrodidyma* causing root rot of olive trees (*Olea europaea*) in California. Plant Dis.

[56] dos Santos RF, Blume E, Muniz MFB, Heckler LI, Finger G, Maciel CG, Harakawa R, Garrido LR (2014). First report of *Ilyonectria macrodidyma* associated with black foot disease of grapevine in Brazil. Plant Dis.

[57] Urakawa R, Shibata H, Kuroiwa M, Inagaki Y, Tateno R, Hishi T, Fukuzawa K, Hirai K, Toda H, Oyanagi N, Nakata M, Nakanishi A, Fukushima K, Enoki T, Suwa Y (2014). Effects of freeze-thaw cycles resulting from winter climate change on soil nitrogen cycling in ten temperate forest ecosystems throughout the Japanese archipelago. Soil Biol Biochem.

[58] Rice AV, Currah RS (2006). Two new species of *Pseudogymnoascus* with *Geomyces* anamorphs and their phylogenetic relationship with Gymnostellatospora. Mycologia.

[59] Lipson DA, Schmidt SK, Monson RK (1999). Links between microbial population dynamics and nitrogen availability in an alpine ecosystem. Ecology.

[60] Pesaro M, Widmer F, Nicollier G, Zeyer J (2003). Effects of freeze-thaw stress during soil storage on microbial communities and methidathion degradation. Soil Biol Biochem.

[61] Walker VK, Palmer GR, Voordouw G (2006). Freeze-thaw tolerance and clues to the winter survival of a soil community. Appl Environ Microbiol.

[62] Larsen KS, Jonasson S, Michelsen A (2002). Repeated freeze-thaw cycles and their effects on biological processes in two arctic ecosystem types. Appl Soil Ecol.

[63] Yashiro E, Pinto-figueroa E, Buri A (2016). Local environmental factors drive divergent grassland soil bacterial communities in the western Swiss alps. Appl Environ Microbiol.

[64] Borg Dahl M, Shchepin O, Schunk C (2018). A four year survey reveals a coherent pattern between occurrence of fruit bodies and soil amoebae populations for nivicolous myxomycetes. Sci Rep.

[65] Margesin R, Jud M, Tscherko D, Schinner F (2009). Microbial communities and activities in alpine and subalpine soils. FEMS Microbiol Ecol.

[66] Robinson PM, Morris GM (1984). Tolerance of hyphae of *Fusarium oxysporum* f.sp. *lycopersici* to low temperature. Trans Br Mycol Soc.

[67] Nieminen JK, Setälä H (2001). Bacteria and microbial-feeders modify the performance of a decomposer fungus. Soil Biol Biochem.

[68] Kuffner M, Hai B, Rattei T, Melodelima C, Schloter M, Zechmeister-Boltenstern S, Jandl R, Schindlbacher A, Sessitsch A (2012). Effects of season and experimental warming on the bacterial community in a temperate mountain forest soil assessed by 16S rRNA gene pyrosequencing. FEMS Microbiol Ecol.

[69] Anderson OR (2016). Experimental evidence for non-encysted, freeze-resistant stages of terrestrial naked amoebae capable of resumed growth after freezethaw events. Acta Protozool.

[70] Graumann P, Schröder K, Schmid R, Marahiel MA (1996). Cold shock stress-induced proteins in *Bacillus subtilis*. J Bacteriol.

[71] Tibbett M, Sanders FE, Cairney JWG (2002). Low-temperature-induced changes in trehalose, mannitol and arabitol associated with enhanced tolerance to freezing in ectomycorrhizal basidiomycetes (*Hebeloma* spp.). Mycorrhiza.

[72] Bardgett RD, Manning P, Morriën E, de Vries FT (2013). Hierarchical responses of plant-soil interactions to climate change: consequences for the global carbon cycle. J Ecol.

[73] van der Putten WH, Bardgett RD, Bever JD, Bezemer TM, Casper BB, Fukami T, Kardol P, Klironomos JN, Kulmatiski A, Schweitzer JA, Suding KN, van de Voorde TFJ, Wardle DA (2013). Plant-soil feedbacks: the past, the present and future challenges. J Ecol.

[74] Kilpeläinen J, Vestberg M, Repo T, Lehto T (2016). Arbuscular and ectomycorrhizal root colonisation and plant nutrition in soils exposed to freezing temperatures. Soil Biol Biochem.

[75] Hasselwandter K, Read DJ (1980). Fungal associations of roots of dominant and sub-dominant plants in high-alpine vegetation systems with special reference to mycorrhiza. Oecologia.

[76] Newsham KK, Upson R, Read DJ (2009). Mycorrhizas and dark septate root endophytes in polar regions. Fungal Ecol.

[77] Vohník M, Albrechtová J (2011). The co-occurrence and morphological continuum between ericoid mycorrhiza and dark septate endophytes in roots of six european *Rhododendron* species. Folia Geobot.

[78] Deslippe JR, Hartmann M, Mohn WW, Simard SW (2011). Long-term experimental manipulation of climate alters the ectomycorrhizal community of *Betula nana* in Arctic tundra. Glob Chang Biol.

[79] Hoeksema JD, Chaudhary VB, Gehring CA, Johnson NC, Karst J, Koide RT, Pringle A, Zabinski C, Bever JD, Moore JC, Wilson GWT, Klironomos JN, Umbanhowar J (2010). A meta-analysis of context-dependency in plant response to inoculation with mycorrhizal fungi. Ecol Lett.

[80] Gryndler M, Hršelová H, Vosátka M, Votruba J, Klír J (2001). Organic fertilization changes the response of mycelium of arbuscular mycorrhizal fungi and their sporulation to mineral NPK supply. Folia Microbiol.

[81] Floss DS, Levy JG, Levesque-Tremblay V, Pumplin N, Harrison MJ (2013). DELLA proteins regulate arbuscule formation in arbuscular mycorrhizal symbiosis. Proc Natl Acad Sci.

[82] Treseder KK (2004). A meta-analysis of mycorrhizal responses to nitrogen, phosphorus, and atmospheric CO_2_ in field studies. New Phytol.

[83] IPCC (2013) Summary for Policymakers. In: Stocker TF, Qin D, Plattner GK, Tignor M, Allen SK.Climate Change 2013: The physical science basis. Contribution of working group I to the fifth assessment report of the Intergovernmental Panel on Climate Changee. Intergov panel Clim Chang

