# Supplementary material for: Root-Associated Mycobiomes of Common Temperate Plants (Calluna vulgaris and Holcus lanatus) Are Strongly Affected by Winter Climate Conditions
Source: Microb Ecol. 2021 Jan 16;82(2):403–15. doi: 10.1007/s00248-020-01667-7 (PMC8384817; doi:10.1007/s00248-020-01667-7)

Suppl. S3 for the article by Mathilde Borg Dahl (dahlm (at) uni-greifswald.de), Derek Persoh, Anke Jentsch and Jürgen Kreyling. Root-associated mycobiomes of common temperate plants (*Calluna vulgaris* and *Holcus lanatus*) are strongly affected by winter. climate conditions Microbial Ecology

OTU accumulation curves - All

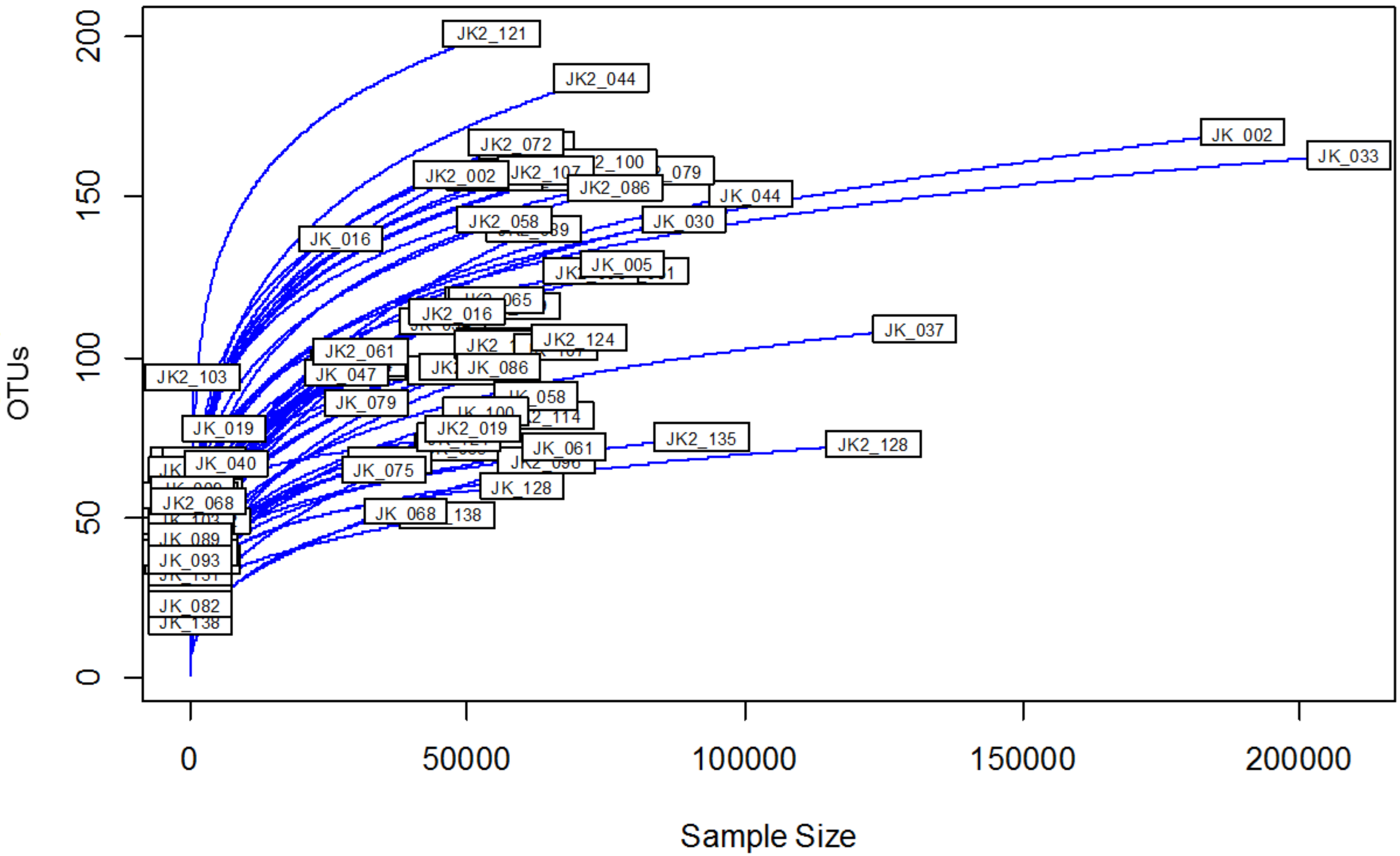

OTU accumulation curves – for inspection

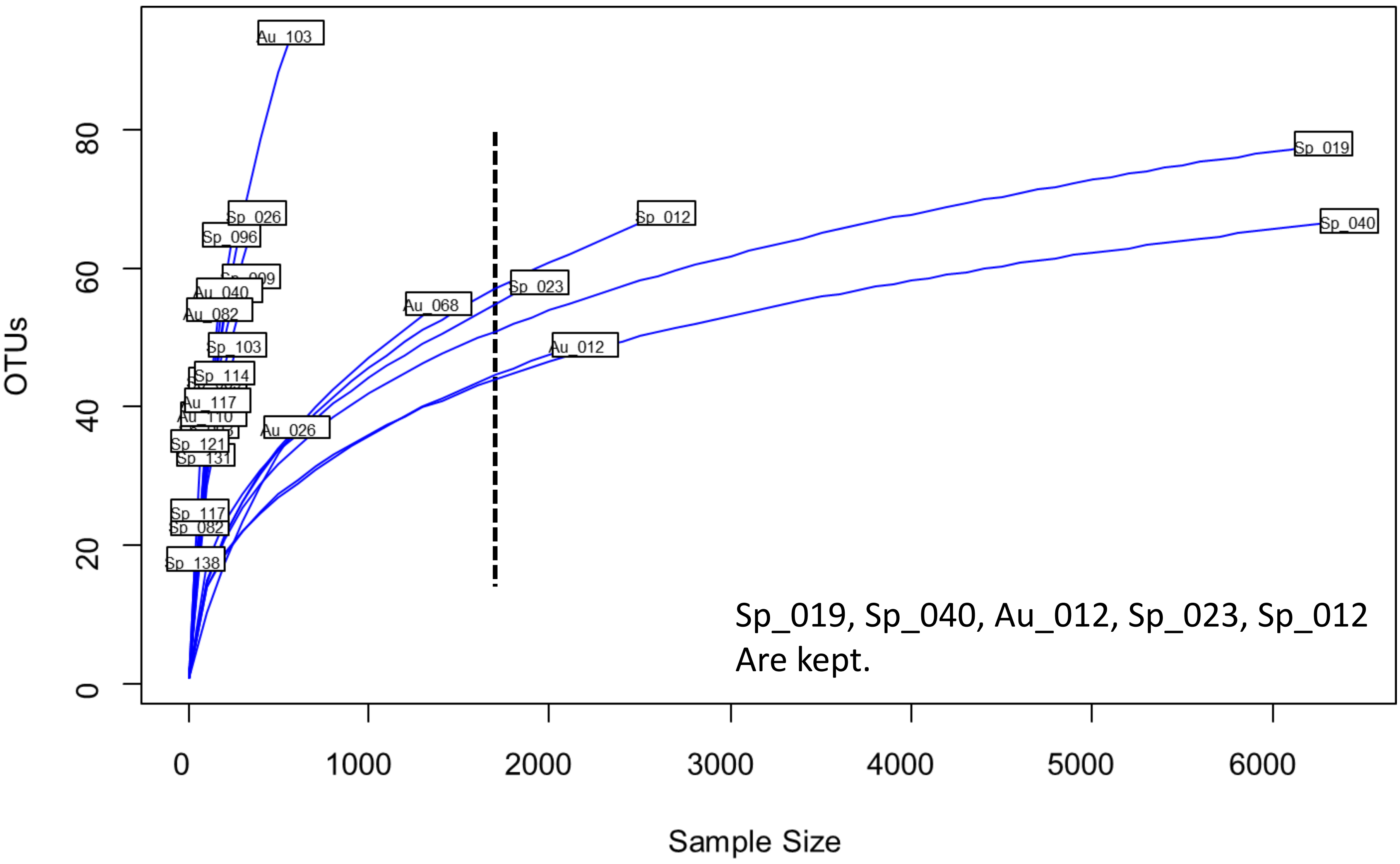

Excluded samples

Site<br>Low/Up	Treatment<br>Warming/Ambient	Sampling<br>Spring/Fall
5	14	14
16	7	7
**Sum**		**21**

Sample-based accumulation curves - All

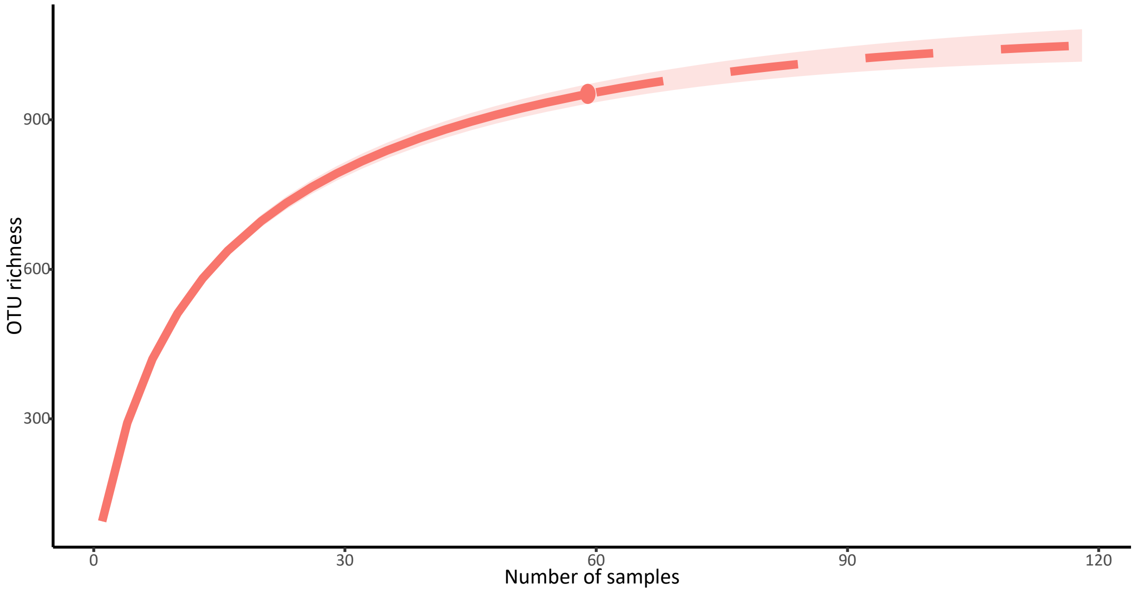

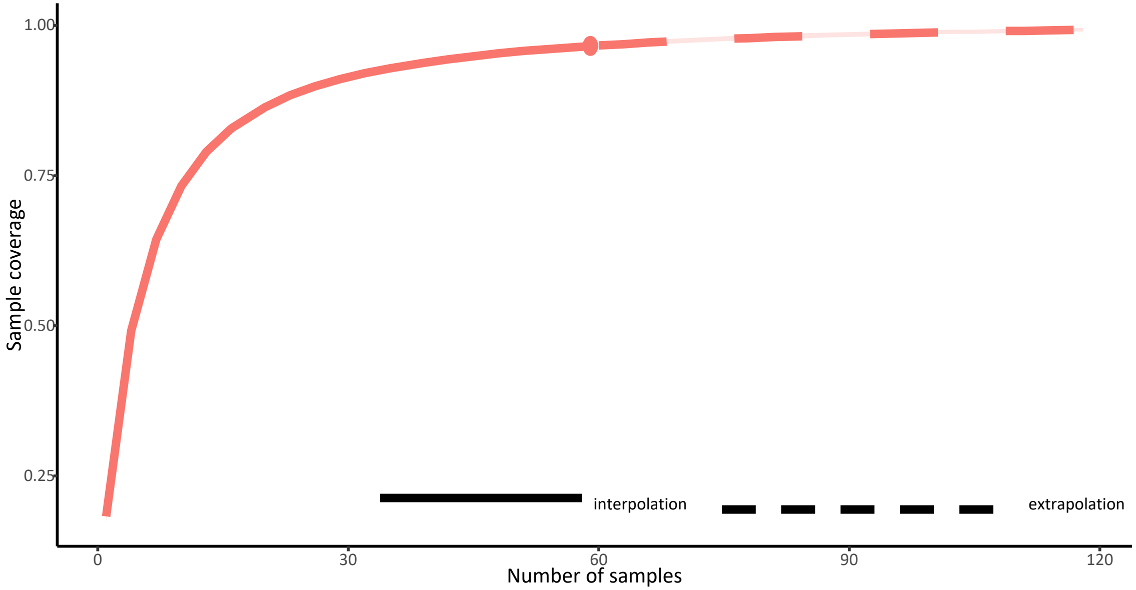

Sample-based accumulation curves – between plants

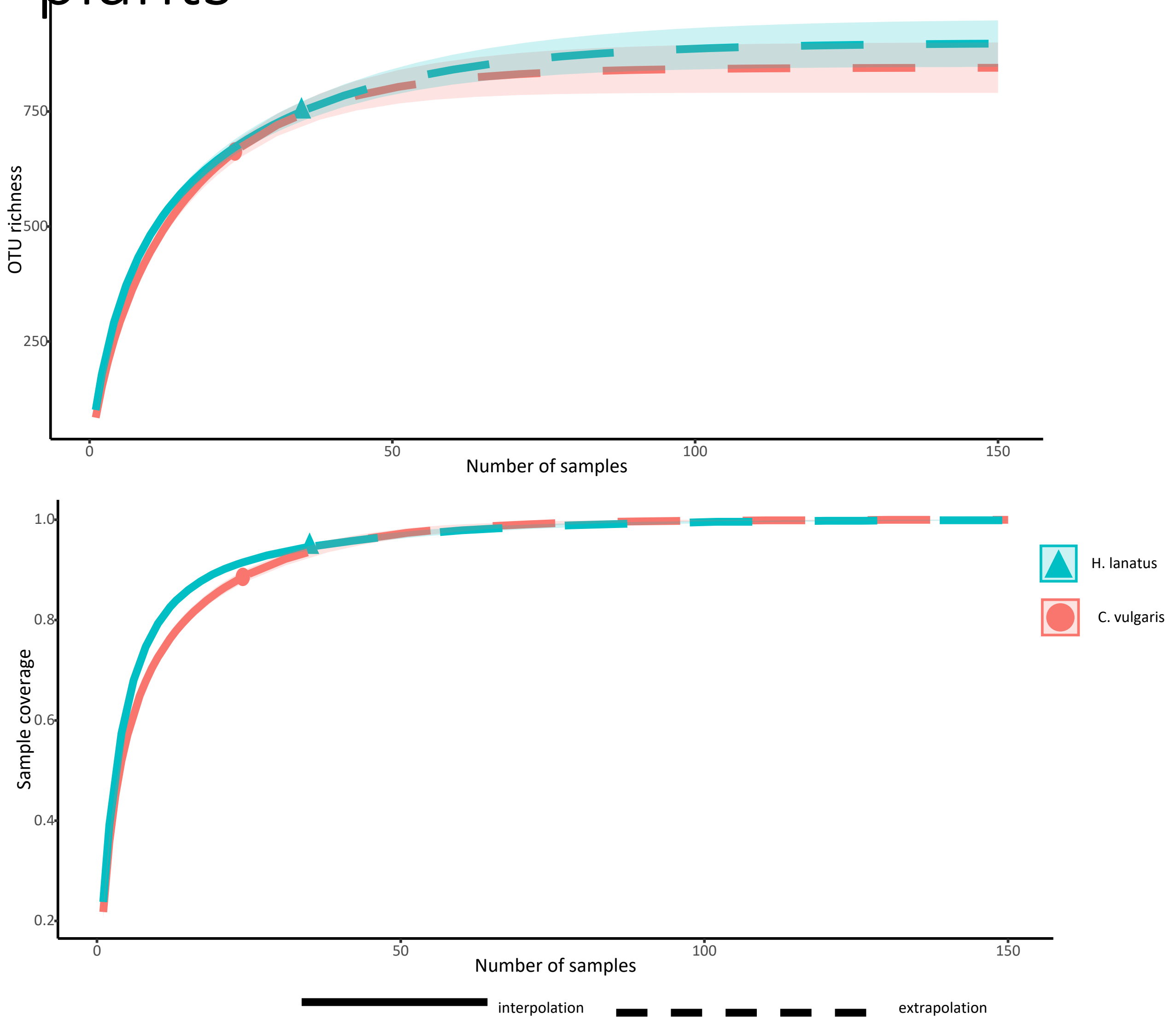

Supplement: Supplementary file 3 — DNA- and sample-based OTU accumulation curves. (PDF 253 kb) [file 248_2020_1667_MOESM3_ESM.pdf]
