# Supplementary figures and images for: Root-Associated Mycobiomes of Common Temperate Plants (Calluna vulgaris and Holcus lanatus) Are Strongly Affected by Winter Climate Conditions
Source: Microb Ecol. 2021 Jan 16;82(2):403–15. doi: 10.1007/s00248-020-01667-7 (PMC8384817; doi:10.1007/s00248-020-01667-7)

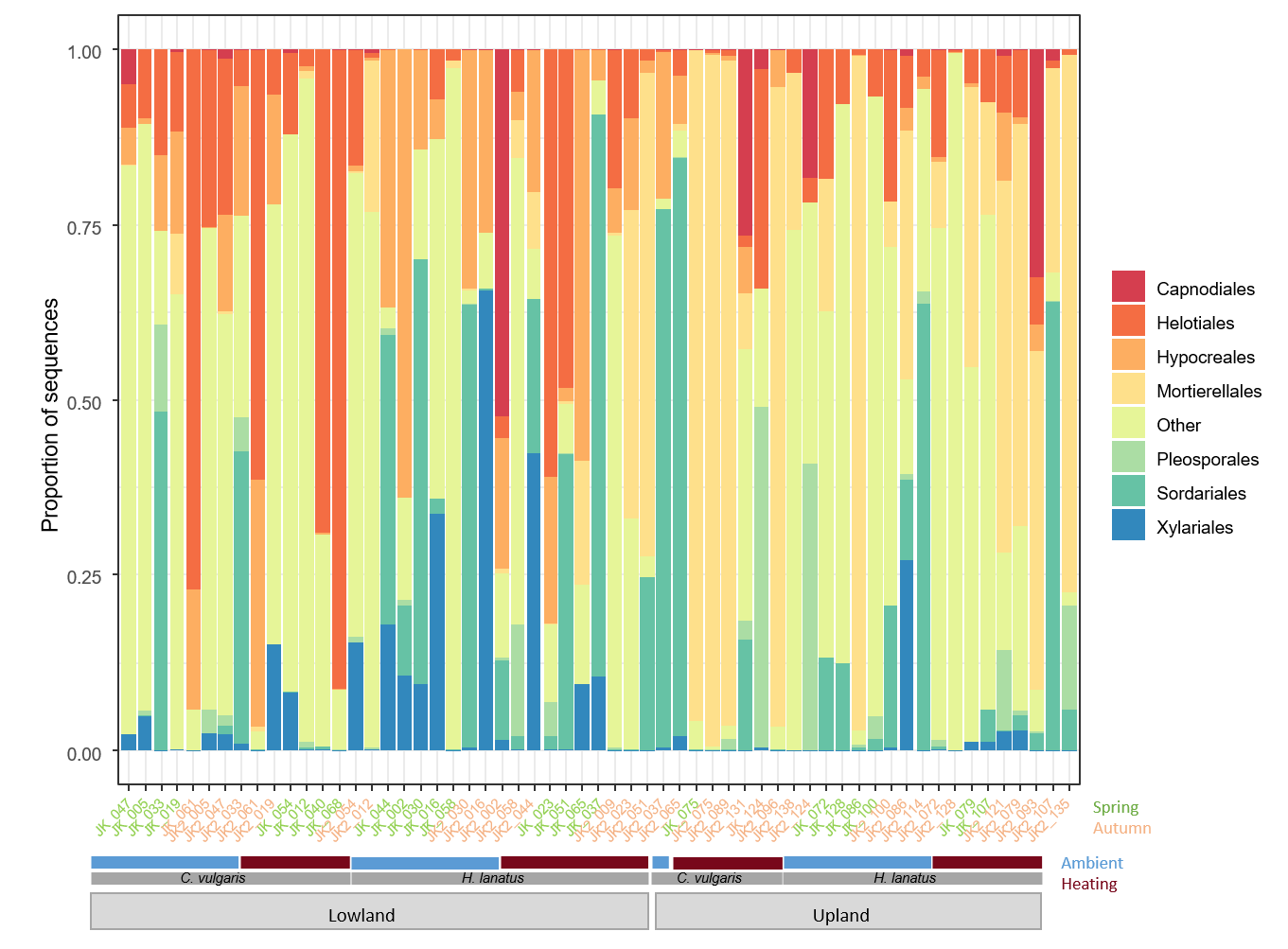

Supplement: Supplementary file 6 — Bar chart showing individual sample variance. (PNG 240 kb) [file 248_2020_1667_MOESM6_ESM.png]
