# Supplementary material for: Root-Associated Mycobiomes of Common Temperate Plants (Calluna vulgaris and Holcus lanatus) Are Strongly Affected by Winter Climate Conditions
Source: Microb Ecol. 2021 Jan 16;82(2):403–15. doi: 10.1007/s00248-020-01667-7 (PMC8384817; doi:10.1007/s00248-020-01667-7)

Suppl. S7 for the article by Mathilde Borg Dahl (dahlm (at) uni-greifswald.de), Derek Persoh, Anke Jentsch and Jürgen Kreyling. Root-associated mycobiomes of common temperate plants (*Calluna vulgaris* and *Holcus lanatus*) are strongly affected by winter climate conditions. Microbial Ecology.

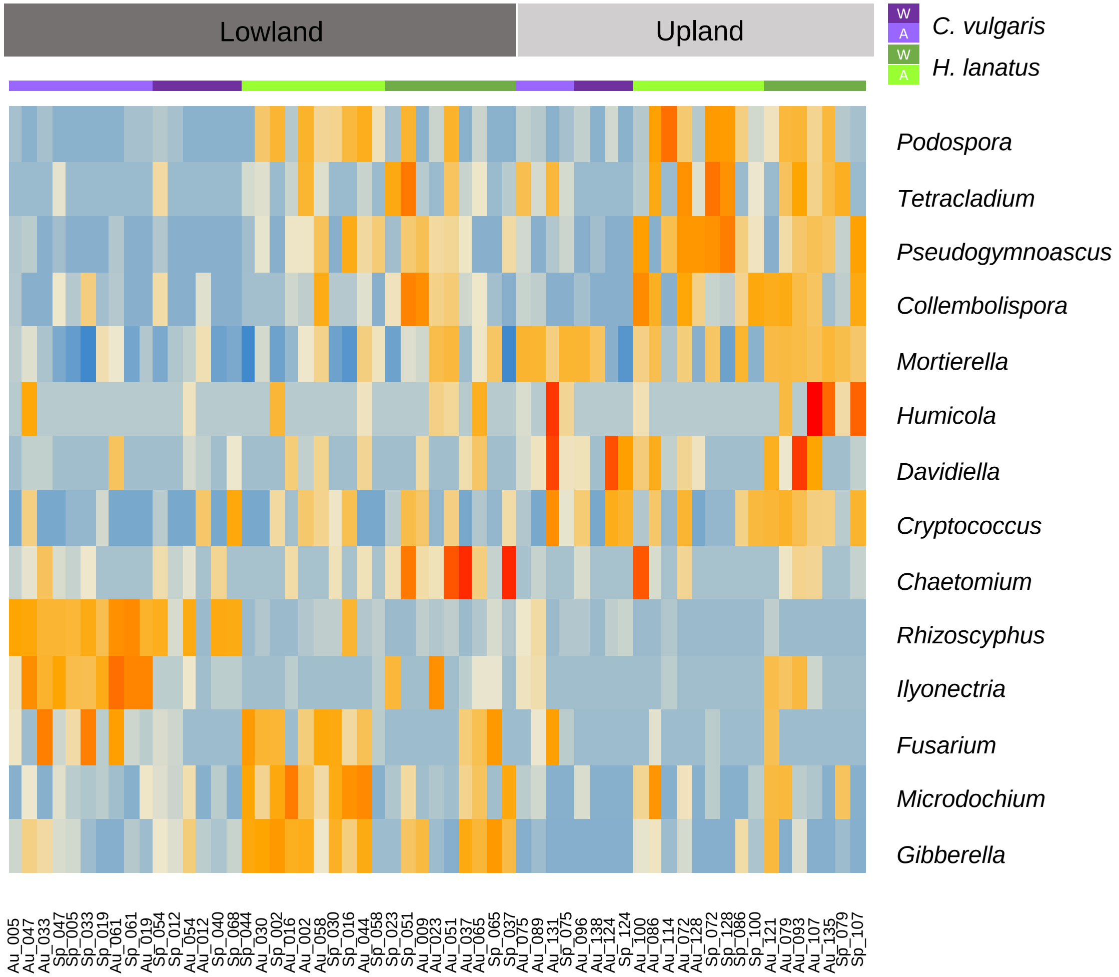

Supplement: Supplementary file 7 — Heatmap of summarized community (genus level). (PDF 132 kb) [file 248_2020_1667_MOESM7_ESM.pdf]
